# Air-Liquid Exchange by Free Hand and One Needle for Unhealed Macular Hole

**DOI:** 10.1155/2022/3052366

**Published:** 2022-01-31

**Authors:** Haishuang Lin, Yiting Ji, Luqi Xia, Bo Lin, Zhihua Li, Zhixiang Hu, Ronghan Wu

**Affiliations:** ^1^School of Ophthalmology and Optometry, Wenzhou Medical University, Wenzhou 325027, Zhejiang Province, China; ^2^Eye Hospital of Wenzhou Medical University, Wenzhou 325027, Zhejiang Province, China

## Abstract

**Aim:**

To report the treatment of 7 cases of unsealed hole after macular hole surgery with air-fluid exchange.

**Methods:**

Retrospective case series. We collected 7 eyes of 7 patients with unsealed hole an unsealed hole about 2 weeks after macular hole surgery (23G vitrectomy with internal limiting membrane peeling with sterilizing air tamponade) in our hospital from February 2018 to December 2018. All patients underwent “air-liquid exchange by free hand and one needle.” The prone position was taken one week after operation. The macular holes before and after operation were examined by frequency-domain optical coherence tomography (SD-OCT).

**Results:**

The size of the macular hole before vitrectomy was 481 ± 156 *μ*m (range: 281–609 *μ*m). Two weeks after vitrectomy (before air and liquid exchange), the size of the macular hole was 295 ± 92 *μ*m (range: 210–421 *μ*m). All macular holes were closed within 7–14 days after air-liquid exchange. There was no complaint of discomfort among these patients.

**Conclusion:**

From this preliminary study, air-liquid exchange by free hand and one needle seems to be safe and effective in the treatment for patients with unsealed and tiny macular hole after vitrectomy as the lack of long effective gas in China. However, the exact efficacy and safety need further large case studies.

## 1. Introduction

Vitrectomy with internal limiting membrane (ILM) peeling with inflating gas tamponade is the first choice for the treatment of idiopathic macular hole (MH), with a closure rate ranging from 68% to 98% [1, 2]. Some studies show that sterilizing air can also be used for macular hole repair and achieve satisfactory results [3–7]. The advantage of sterilizing air is that the time of prone position is short [8], which is not easy to cause high intraocular pressure. However, the disadvantage is that the absorption time of sterilizing air is short so that the closure rate in the large size of macular hole is lower than inflating gas [9]. Reoperation or gas-liquid exchange with inflating gas is usually used for unsealed hole after macular hole surgery. The operation procedure is complicated which may increase the risk of infection and the burden of patients. Therefore, we report a case series of unsealed hole after macular hole surgery. Patients subsequently underwent “air-liquid exchange by free hand and one needle” with sterilizing air.

## 2. Subjects/Materials and Methods

### 2.1. Subjects

This case series was retrospective and included 7 eyes from 7 patients with unsealed hole about 2 weeks after macular hole surgery (23G vitrectomy with ILM peeling with sterilizing air tamponade) at the Eye Hospital of Wenzhou Medical University from February 2018 to December 2018.

The inclusion criteria included patients with unsealed hole about 2 weeks after macular hole surgery, and spectral domain optical coherence tomography (OCT) (Spectralis Hra Oct; Heidelberg Engineering, Heidelberg, Germany) showed that the diameter was less than 450 *μ*m. Exclusion criteria included patients with glaucoma, macular degenerative disease, ocular trauma, ocular tumors, and optic atrophy. Observations regarding macular hole were carried out using the SD-OCT. Data obtained included age, gender, affected eye, MH size before 23G vitrectomy, MH size before air-liquid exchange, MH closure time, and uncorrected visual acuity.

### 2.2. Surgical Procedure

**Figure 1:**
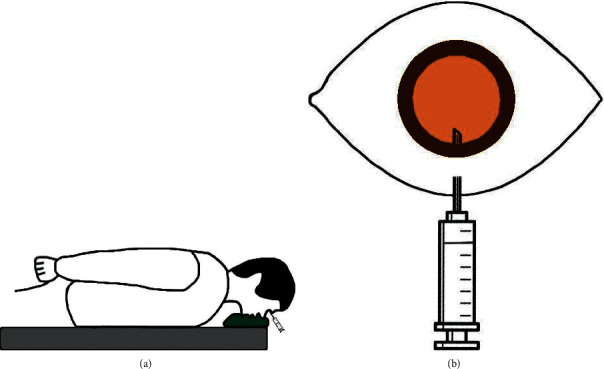
Schematic diagram of “air-liquid exchange by free hand and one needle.” (a) Patient took the prone position; (b) the needle was inserted obliquely at 3.5 mm of the inferior limbus.

All patients underwent “air-liquid exchange by free hand and one needle” under topical anesthesia. The patient took the supine position. Anterior chamber penetration was performed to decrease the intraocular pressure into Tn-1 after the conjunctival sac was washed. Then, the patient changed to the prone position. After that, the operating table was raised to the highest position. The patient slightly raised his head and fixed the eye on the supernasal side. Subsequently, 5 ml of sterilizing air was drawn off with a 5 ml syringe, and a 28 G air injection needle was replaced. The needle was placed at the inferior side 3.5 mm away from the limbus and obliquely inserted into the ocular about 7 mm (shown in Figure 1(a)). The sterilizing air was injected with vitreous humor was drawn off. Finally, the partial needle was pulled out slowly, and the intraocular pressure was roughly measured by finger sense of the surgeon (shown in Figure 1(b)).

The above-mentioned steps were repeated until the air was observed behind the intraocular lens of the pupil area, and the liquid flow was no longer seen. At this time, the vitreous cavity was filled with air, and the liquid can no longer be drawn off. The liquid drawn off from the vitreous cavity was about 4 ml. Subsequently, the needle was pulled out quickly, and the cotton swab was pressed on the puncture port for 3 min. When the needle was pulled out completely, no vitreous humors flowed out. The conjunctival sac was washed again after the patient changed to the supine position. Anterior chamber drainage was performed if intraocular pressure was high. The patients were asked to remain a prone position and to avoid the supine position for one week. All surgeries were performed by one experienced surgeon (Hu ZX).

## 3. Results

**Figure 2:**
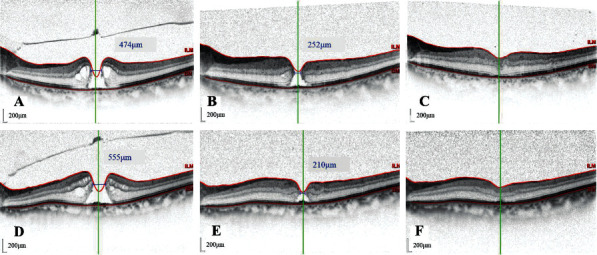
The size of macular holes in three patients before and after surgery. A–C: the size of the macular hole before vitrectomy, before gas-liquid exchange in patient 5, and the macular hole closed 7 days after gas-liquid exchange; D–F: the size of the macular hole before vitrectomy, before gas-liquid exchange in patient 6, and the macular hole closed 7 days after gas-liquid exchange.

Our “air-liquid exchange by free hand and one needle” technique was performed in 7 patients. The age of 7 patients was 65.4 ± 6.8 years, of which three were male. The size of the macular hole before vitrectomy was 481 ± 156 *μ*m (range: 281–609 *μ*m). Two weeks after vitrectomy (before air and liquid exchange), the size of the macular hole was 295 ± 92 *μ*m (range: 210–421 *μ*m) (shown in Table 1). All macular holes were closed within 7–14 days after air-liquid exchange (shown in Figure 2). The postoperative uncorrected visual acuity had improved from 1.47 ± 0.59 logarithm of the minimum angle of resolution (logMAR) units preoreratively to 0.98 ± 0.30 logMAR units at 1-month visit after air-liquid exchange (*t* = 3.131, *P*=0.02) (shown in Table 1). The visual acuity of six patients (85.7%) improved. There was no complaint of discomfort within these patients in the 1-month follow-up examinations. None of the eyes has required subsequent surgical procedures.

## 4. Discussion

The effective treatment for idiopathic macular hole with smaller diameter (within 600 *μ*m) is vitrectomy with ILM peeling with inflating gas tamponade. The success rate of the operation is more than 90% [1]. However, sterilizing air is the main tamponade for the treatment of macular hole, since inflating gas is unavailable in China. Compared with inflating gas, the absorption of sterilizing air is quicker (average 9 days) [10].

There are few studies that compared the effects of sterilized air and inflating gas tamponades on recovery after vitrectomy for the treatment of idiopathic macular hole. He F et al. pointed out that the primary closure rate was 90.6% in the sterilized air group and 95.0% in the perfluoropropane (C3F8) group one month after surgery. There was no statistically significant difference in the closure rate. Also, they concluded that vitrectomy with sterilized air tamponade is safe and effective for the treatment of idiopathic macular hole [9]. Usui H et al. suggested that air tamponade provided a comparable rate of MH closure compared with SF6 gas tamponade, at least for MH with preoperative diameter smaller than 500 µm [11]. Forsaa VA et al. proposed that, in the group of full-thickness macular hole ≤400 µm, primary closure occurred in 95%, whereas only 57% of those >400 µm closed [10].

For patients with unsealed hole after macular hole surgery, revitrectomy with transplantation of residual ILM or other tissue with silicone oil or gas tamponade is usually adopted [12–14]. However, the operation procedure is complicated which may increase the risk of infection. Rao X reported that 89% patients achieved anatomic success after the outpatient inflating gas-fluid exchange by two needles for open macular hole after primary vitrectomy [15, 16]. Additional intravitreal inflating gas injection can achieve complete macular hole closure for unsealed macular hole ≤700 µm [17, 18].

In this case series, we treated 7 eyes from 7 patients with an unhealed macular hole within 450 *μ*m. Complications such as endophthalmitis, high intraocular pressure, vitreous hemorrhage, iatrogenic retinal tear, or retinal detachment have not been observed in the 1-month follow-up. The macular hole of all patients was closed within two weeks after “air-liquid exchange by free hand and one needle.” None of the eyes has required subsequent surgical procedures. Compared with two needles, the one needle technique of our case series is easy to operate with controllable intraocular pressure and low risk of infection, which can be completed in the outpatient operating room.

Gas tamponade can restrict the flow of fluid from the vitreous cavity to the macular region, keep the macular region dry, and promote the proliferation and transplantation of glial cells [19]. By additional gas injection, the surface of the macular hole can be kept dry for one week, which can activate the unhealed macular hole to reproliferate and repair. The closure of the macular hole can be achieved by additional gas injection even if the macular hole is not closed in the primary macular hole surgery [20–22].

In conclusion, this preliminary study suggests that the new technique “air-liquid exchange by free hand and one needle” using sterilizing air obtained positive results and seems to be safe for eyes with unhealed holes. Long-term anatomic and functional recovery in patients with “air-liquid exchange by free hand and one needle” merit further study.

## Figures and Tables

**Table 1:** Baseline characteristics and postoperative surgical outcome of each patient.

Cases	Gender/age	Operated eye	Preoperative visual acuity	Size of MH before vitrectomy (*μ*m)	Size of MH two weeks after vitrectomy (*μ*m)	MH closure time (days)	Postoperative visual acuity at 1 month	Complications
1	F/62	Right	FC/40 cm	609	345	9	0.05	None
2	F/71	Right	0.04	567	401	9	0.2	None
3	M/53	Right	FC/10 cm	502	421	9	0.05	None
4	F/64	Left	0.05	379	223	14	0.05	None
5	M/73	Left	0.08	474	252	7	0.2	None
6	M/70	Left	0.1	555	210	7	0.16	None
7	F/65	Right	0.12	281	214	11	0.16	None

## Data Availability

The data used to support this study can be obtained from the corresponding author on request.
